# Intra-observer reproducibility and inter-observer agreement of Fels skeletal age assessments among male tennis players 8–16 years

**DOI:** 10.1186/s12887-023-03965-8

**Published:** 2023-04-26

**Authors:** Paulo Sousa-e-Silva, Manuel J. Coelho-e-Silva, Jorge M. Celis-Moreno, Daniela C. Costa, Diogo V. Martinho, Luís P. Ribeiro, Tomas Oliveira, João Gonçalves-Santos, Oscar M. Tavares, Joaquim M. Castanheira, Telmo Pereira, Jorge Conde, Ricardo R. Cayolla, Pedro Duarte-Mendes, Gillian K. Myburgh, Sean P. Cumming, Robert M. Malina

**Affiliations:** 1 University of Coimbra, FCDEF, Coimbra, Portugal; 2 University of Coimbra, CIDAF (uid/04213/2020), Coimbra, Portugal; 3 Universidad Santo Tomás, Bogotá, Colombia; 4 University of Algarve, School of Health, Faro, Portugal; 5 Polytechnic Institute of Coimbra, Coimbra Health School, Coimbra, Portugal; 6 University Portucalense, REMIT (Research Centre on Economics, Management and Information Technologies), Porto, Portugal; 7 Polytechnic Institute of Castelo Branco, School of Education, Castelo Branco, Portugal; 8 Polytechnic Institute of Castelo Branco, Sport, Health & Exercise Research Unit (SHERU), Castelo Branco, Portugal; 9 Fulham FC, London, UK; 10 University of Bath, Department of Health, Bath, UK; 11 University of Texas at Austin, Department of Kinesiology and Health Education, Austin, TX USA; 12 University of Louisville, School of Public Health and Information Sciences, Louisville, KY USA

**Keywords:** Youth sports, Biological maturation, Bone age, Skeletal maturation, Maturity status

## Abstract

**Background:**

Skeletal age (SA) is an estimate of biological maturity status that is commonly used in sport-related medical examinations. This study considered intra-observer reproducibility and inter-observer agreement of SA assessments among male tennis players.

**Methods:**

SA was assessed with the Fels method in 97 male tennis players with chronological ages (CA) spanning 8.7–16.8 years. Radiographs were evaluated by two independent trained observers. Based on the difference between SA and CA, players were classified as late, average or early maturing; if a player was skeletally mature, he was noted as such as an SA is not assigned.

**Results:**

The magnitude of intra-individual differences between repeated SA assessments were d = 0.008 year (observer A) and d = 0.001 year (observer B); the respective coefficients of variation were 1.11% and 1.75%. Inter-observer mean differences were negligible (t = 1.252, *p* = 0.210) and the intra-class correlation coefficient was nearly perfect (ICC = 0.995). Concordance of classifications of players by maturity status between observers was 90%.

**Conclusion:**

Fels SA assessments were highly reproducible and showed an acceptable level of inter-observer agreement between trained examiners. Classifications of players by skeletal maturity status based on assessments of the two observers were highly concordant, though not 100%. The results highlight the importance of experienced observers in skeletal maturity assessments.

**Supplementary Information:**

The online version contains supplementary material available at 10.1186/s12887-023-03965-8.

## Background

Biological maturation is a process marking progress toward the biologically mature or adult state. Although assessments of skeletal age (SA) based on the bones of the hand and wrist are generally used in a clinical context, SA is also a commonly used indicator of maturity status in studies of youth athletes [1, 2]. An assigned SA is the chronological age (CA) at which specific stages of maturity of the hand-wrist bones are attained relative to the reference sample upon which the method of assessment was developed. Three methods are often used to estimate SA [3]: Greulich-Pyle (GP) [4], Tanner-Whitehouse (TW) [5–7] and Fels [8]. Although the methods specific to the three protocols vary, each requires a radiograph of the hand-wrist and is based on the universal and invariant sequences of development of each bone from initial calcification to the mature state.

Although automated protocols for the assessment of radiographs have recently been implemented [9, 10] assessment of SA is generally done by experienced examiners. Studies, however, do not ordinarily report intra-examiner and inter-examiner variability in assessments. Nevertheless, a broad range of normal variability is commonly accepted in the clinical context. Studies reporting variability within and between assessors of SA have commonly used relatively small samples for replicate assessments. Several studies reporting Fels SAs in youth athletes noted relatively small mean differences within and between observers and also small technical errors of measurements [11–14]. Information on intra- and inter-observer variability in Fels SA assessments in larger samples and also in assigned stages of specific bones is lacking. The potential influence of observer-related error associated with SA assessments is also of relevance in applications of SA in CA verification [15, 16] and also in adapting training protocols for youth athletes based on maturity status [17, 18]. In the context of the preceding, the purpose of this study is to determine intra- and inter-observer reproducibility of assessments of bone-specific maturity indicators and assigned SAs with the Fels method in a relatively large sample of male tennis players (*n* = 97). Radiographs were assessed by two observers within an interval of 1 month.

## Methods

### Study design and participants

The procedures for the present study followed ethical standards established for sports sciences [19]. The project was approved by the *Ethics Committee for Sports Sciences* by the *University of Coimbra* (CE/FCDEF-UC/00122014). Parents of each player provided informed consent. Players were informed about the objectives, procedures, benefits and risks of the project and also that they could withdraw from the study at any time. The sample included 97 male tennis players ranging in CA from 8.7–16.8 years. CA was calculated as the difference between birth date and the date of the visit to the clinic for the radiograph. The players trained for at least two seasons under the supervision of a certified coach at a tennis club, and also competed in official tournaments organized by the national tennis federation.

### Determination of skeletal age

A posterior-anterior radiograph of the left hand-wrist was available for each player. SA was estimated with the Fels method [8] which requires the evaluation of 22 bones: radius, ulna, seven carpals (capitate, hamate, triquetral, lunate, scaphoid, trapezium, trapezoid), three metacarpals (I, III, V), proximal and distal phalanges of three digits (I, III, V), middle phalanges of digits III and V, and the absence or presence of the pisiform and adductor sesamoid. Evaluations are based on specific criteria for each bone, i.e., the presence or absence of the ossification centre, changes in shape, radiopaque lines, and epiphyseal-diaphyseal fusion of the long bones and attainment of adult morphology for the carpals. Measurements of epiphyseal and diaphyseal widths of the long bones are also required. The grades assigned for each bone, and the epiphyseal and metaphyseal widths are entered into a computer program (Felshw 1.0) that calculates the SA and the standard error of estimate for the individual. CA was subtracted from the SA of each player (SA minus CA). Based on the SA-CA difference, each participant was classified as: late (SA < CA by more than 1.0 year), on time or average (SA within ± 1.0 year of CA), or early (SA > CA by more than 1.0 year) maturing. If a player had attained skeletal maturity, an SA was not assigned and he was simply indicated as skeletally mature. The band ± 1.0 year is commonly used in samples of youth, both non-athletes and athletes [3]. The band accommodates variation in SA per se and also variation associated with error in assessments. All radiographs were independently evaluated by two experienced individuals (observer A and observer B) on two occasions (time-moment 1; time-moment 2). Both observers were experienced in the estimation of SA using the Fels method; each had completed more than 1000 examinations over the past few years [20, 21].

#### Analyses

Frequencies of intra-observer error by bone were calculated separately for the two examiners. Technical errors of measurement (TEM) and coefficients of variation (%CV) were also determined. Mean differences between time moments were evaluated using paired t-tests. In addition, paired t-tests comparing inter-observer agreement and intra-class correlation coefficients (ICC) were calculated for time-moment 2. The magnitude of effects was evaluated with Cohen d-values [22] as follows: d < 0.20 (trivial), 0.20 < d < 0.60 (small), 0.60 < d < 1.20 (moderate), 1.20 < d < 2.00 (large), 2.00 < d < 4.00 (very large), and > 4.00 (nearly perfect) [23]. Finally, the limits of agreement between observers were obtained using Bland–Altman analysis [24]. The concordance of maturity status classifications of the two observers based on the difference of SA – CA was determined for the total sample. As noted, players who were skeletally mature were not included in the analyses. One player was rated as skeletally mature by observer A (in time-moment 1 and also in time-moment 2), while four players were evaluated as skeletally mature by observer B in time-moment 2 (two were already classified as mature in time-moment 1). The sample size for the analysis was thus 96 for intra-observer agreement by examiner A; 93 non-skeletally mature players for intra-observer agreement by examiner B and inter-observer agreement at time-moment 2. The Statistical Package for the Social Sciences version 26.0 (SPSS Inc., IBM Company, Armonk, NY, USA) and GraphPad Prism (version 5 for Windows, GraphPad Software, San Diego California USA, www.graphpad.com) were used in the analyses. Significance level was set at *p* < 0.05.

## Results

Descriptive statistics for estimates of Fels SAs for observers A and B at each time moment are summarized in Table 1. Intra-individual mean differences and coefficients of variation were negligible. Overall, the two observers accumulated 605 errors in the replicate assessments of individual bones (observer A: 234, observer B: 371; see Table 2). Note, that number of criteria for each bone varies. Based on the summed frequencies of assessment errors of the two observers, specific indicators of four bones were most problematic: 29 in metacarpal III (indicator MET III-4: proximo-medial projection of the epiphysis), 28 in the trapezoid (indicator TPD-3: shape of the medial margin of the trapezoid), 22 in the triquetral (indicator TRI-2: shape of the lateral margin of the triquetral), and 22 in the trapezium (indicator TPM-4: radiopaque line or zone within the proximal margin of the trapezium).

**Table 1:** Descriptive statistics (means ± standard deviations) for skeletal ages of male tennis players (*n* = 97) by time-moment separately for observer A and observer B; results of comparisons between time moments, technical errors of measurement (TEM) and coefficients of variation (%CV)

Examiner	Unit	Time moment		Paired t- test		Magnitude effect		Reliability	
		TM1	TM2	t	*p*	d	(qualitative)	TEM	%CV
Observer A^a^	years	13.18 ± 2.47	13.18 ± 2.45	0.098	0.922	0.008	(trivial)	0.15	1.11
Observer B^b^	years	13.07 ± 2.39	13.09 ± 2.40	0.563	0.575	0.001	(trivial)	0.23	1.75

*TM1* Time-moment 1, *TM2* Time-moment 2, *d* Cohen d-value, *p* Significance level, *TEM* Technical error of measurement, *%CV* Coefficient of variation

^a^Observer A assigned one participant as skeletally mature and were excluded

^b^Observer B assigned four participants as skeletally mature and were excluded

**Table 2:** Frequencies of intra-observer error by bone

Observer A^a^		Observer B^b^	
Bone	(f)	Bone	(f)
Trapezoid	32	Trapezium	45
Trapezium	28	Metacarpal III	40
Metacarpal V	21	Trapezoid	37
Proximal phalange I	21	Radius	32
Metacarpal III	18	Metacarpal V	31
Proximal phalange III	18	Triquetral	24
Triquetral	16	Proximal phalange I	19
Radius	11	Proximal phalange III	17
Medial phalange III	11	Metacarpal I	15
Metacarpal I	10	Lunate	14
Adductor sesamoid	8	Medial phalange III	12
Scaphoid	7	Scaphoid	12
Medial phalange V	6	Proximal phalange V	11
Distal phalange I	6	Ulna	11
Distal phalange III	6	Distal phalange V	8
Proximal phalange V	4	Medial phalange V	7
Lunate	3	Pisiform	7
Pisiform	3	Distal phalange I	6
Distal phalange V	2	Distal phalange III	6
Capitate	2	Capitate	6
Hamate	1	Hamate	6
Ulna	0	Adductor sesamoid	5
	234		371

*f* Absolute frequencies

^a^observer A assigned one participant as skeletally mature and were excluded

^b^observer B assigned four participants as skeletally mature and were excluded

**Fig. 1:**
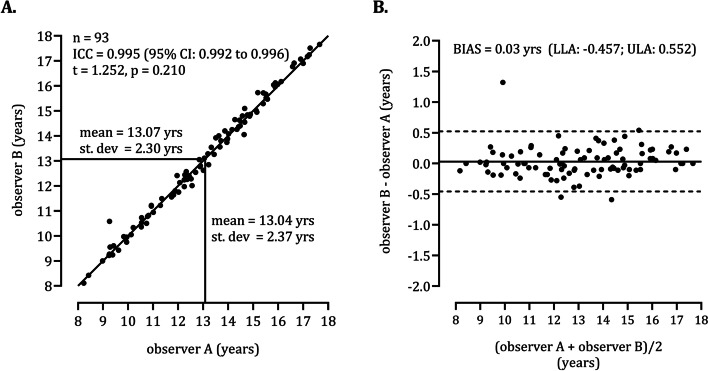
**A** Scatterplot of the SA estimated by Observer B (y-axis) and Observer 2 (x-axis); **B** Discrepancies between observers plotted against average SA of the two observers (Bland–Altman analysis). Legend: ICC (intra-class correlation coefficient; 95% CI (95% confidence intervals); LLA (lower limit of agreement); ULA (upper limit of agreement)

SAs of individual players assigned by observer A (x-axis) were plotted relative to those assigned by observer B (y-axis) in Fig. 1, panel A; the panel also includes the respective means and standard deviations by observer in time moment 2. The mean difference between observers was not significant (t = 1.252, *p* = 0.210), while the ICC was nearly perfect (ICC = 0.995). The Bland–Altman analysis of the intra-individual differences (y-axis) relative to the mean of both observers (x-axis) is illustrated in Fig. 1, panel B. Only four cases exceeded the limits of agreement (lower limit of agreement: -0.457 year; upper limit of agreement: 0.552 year).

Results of the cross-tabulations of maturity status classifications based on assessments of observers A and B for the total sample are summarized in Table 3. The overall agreement between the two observers in time-moment 2 was 90%, i.e., 87 of the total sample of 97 players were classified in the same maturity category by the two examiners.

**Table 3:** Cross-tabulations of maturity status classifications based on assessments of observers A and B among male tennis players for the total sample (*n* = 97)

Observer A (Time moment 1)	Maturity Status (Time moment 2)				Total
	Late	Average	Early	Mature	
Late	**10**	1	0	0	11
Average	2	**44**	2	0	48
Early	0	3	**34**	0	37
Mature	0	0	0	**1**	1
Total	12	48	36	1	97
Observer B (Time moment 1)	Maturity Status (Time moment 2)				Total
Late	**8**	3	0	0	11
Average	3	**45**	4	1	53
Early	0	1	**29**	1	31
Mature	0	0	0	**2**	2
Total	11	49	33	4	97
Maturity Status (Observer A)^a^	Maturity Status (Observer B)^a^				Total
	Late	Average	Early	Mature	
Late	**10**	2	0	0	12
Average	1	**44**	1	2	48
Early	0	3	**32**	1	36
Mature	0	0	0	**1**	1
Total	11	49	33	4	97

Bold values indicate the same maturity status classification with each method of SA assessment

^a^Time-moment 2

## Discussion

SA age is generally accepted as the best method for the estimation of biological maturity status [3]. The present study evaluated intra-observer the reproducibility of SA assessments among 97 male tennis players 8.7–16.8 years. Although both observers were well-experienced in the Fels SA assessment protocol, 234 errors were noted between the assessments of observer A and 371 between the assessments of observer B. By inference, error is part of SA assessment process. Nevertheless, the intra-observer variability had a trivial effect on intra-individual differences in mean SAs. The error associated with each individual observer was also negligible in terms of the %CV. Among the 22 bones assessed by the two observers, five bones – two carpals (trapezium, trapezoid), two metacarpals (III and V) and the radius accounted for 49% of the differences between observations. Nevertheless, the agreement between the two assessors in the present study was acceptable. The plot of the SA assessments of each assessor approached the line of identify (Fig. 1). The differences between observers were not significant and the ICC approached nearly perfection. On the other hand, only 87 of the 97 players were classified as having the same skeletal maturity status (delayed, one time, advanced, mature).

Skeletal age assessment protocols assume a universal and invariant sequence of changes in each of the bones comprising the hand-wrist complex [8]. The rate at which bones progress from the cartilage model to the mature state varies among bones and also within individuals. And as noted, the progress in the process of skeletal maturation can be monitored with standardized radiographs of the hand and wrist, which is comprised of two types of bones: long (distal radius, ulna, metacarpals, and phalanges) and round (carpals, adductor sesamoid). Corresponding protocols have been developed for other regions of the body—knee, foot and ankle, as summarized by Roche et al. [8]. Maturity status based on SA also spans approximately the first two decades of life, which contrasts indicators of sexual maturity status that are limited to the pubertal years. SA also tends to progress independently of dental maturation [3].

Intra-individual variability of SAs based on hand-wrist and knee radiographs was evaluated in a clinical sample (short stature, obesity, acute diseases, short predicted adult height) of 250 girls and 339 boys 2–15 years of age [25]. SA of the knee was based on the Roche-Wainer-Thissen (RWT) protocol, while SA of the hand-wrist was assessed with the GP, TW (TW2-20 bone, TW2 RUS) and Fels methods. SAs based on the knee were, on average, closer to CAs compared to the hand-wrist protocols. Among late maturing individuals (SA < CA by more than 1.5 years), GP, TW2-20 bone, TW2 RUS, and FELS SAs tended to be lower than RWT knee SA estimates. Conversely, among early maturing individuals (SA > CA by more than 1.5 years), estimated hand-wrist SAs tended to be higher than RWT knee SAs. In an earlier clinical study of children and adolescents 1–17 years (171 males, 156 females) hospitalized with clinical pathologies excluding growth disorders [26], the accuracy and precision of FELS, GP and TW2 SAs were considered. Two observers rated the radiographs while one of the observers re-assessed the radiographs after 6 months. The gradient of inter-observer errors expressed as standard errors (*n* = 327) was, respectively, 0.165, 0.203, 0.293 and 0.325 for the Fels, GP and TW2-20 bone and TW2 RUS SAs; the corresponding gradient for standard errors for intra-observer assessments was, respectively, 0.145, 0.170, 0.222 and 0.254. In the present study standard errors were apparently regardless of observer and time-moment. For observer A was 0.309 ± 0.037 year in time-moment 1 and 0.310 ± 0.039 year in time-moment 2. Corresponding means and standard deviations for examiner B were 0.312 ± 0.039 year and 0.306 ± 0.032 year. Note, however, that the magnitude of the standard error increases with chronological age since determination is based on less indicators [8] and consequently caution is needed when interpreting different studies.

In the context of youth sports, few studies have reported the reproducibility of Fels SAs in small samples. Among 15 American football players aged 9–14 years, the intra-observer mean difference was -0.02 [13], while among male adolescent soccer players, replicate assessments of Fels SAs in 15 players indicated a mean difference of 0.08 years [14]. And, repeated assessments of radiographs of 10 soccer players (25% of the sample) by the same observer indicated small differences for Fels SA (0.01 year) and TW3 RUS SA (0.04 year) with TEMs about 0.04 year and 0.06 year, respectively, for each protocol [12]. In the current study, the mean differences between assessments were -0.01 year and 0.02 year, respectively for observer A and observer B, while corresponding TEMs were, respectively, 0.15 year and 0.23 year. Assessments of Fels SAs of 18 U13 and U15 soccer players by two observers indicated a mean difference of 0.03 year [27] which corresponded to the bias noted in the Bland–Altman plot in Fig. 1.

The same protocol was used by two observers in the current study, and skeletal maturity status was misclassified in 10.3% of the players, allowing for the relatively broad band of the SA-CA cut-offs used to define maturity groups. Some degree of error is implicit in SA estimates and the error impacts the distribution of youth by skeletal maturity status. Nevertheless, the classification criteria used in the present study were consistent with other studies of the general population of youth [28] and of athletes [2, 29, 30], although a narrower band (SA-CA difference of ± 3 months) defining early and late maturity status has been used [31]. The latter band, however, is within the range of standard errors of SA assessments.

Studies of variation in maturity status among tennis players are limited, and different indicators of maturity status have been used. Male and female adolescent players advanced in skeletal maturity status (Fels SAs) were, on average, taller, heavier and stronger (grip) than average and late maturing peers, while differences among maturity groups in several performance tasks were variable [30, 32]. A study of potential effects of tennis training frequency on inter-arm bone asymmetry in 24 youth male players self-assessed in stages 1–2 of secondary sex characteristics were grouped according to the number of weekly sessions [33]: 10 participants completed five sessions (10.8 ± 0.7 year), 14 participants completed two sessions (10.4 ± 1.0 year). Although asymmetry of bone mineral content and lean mass in dominant arm was associated to weekly volume of tennis practice, maturity-associated variation in the samples was not considered. The literature suggests training-related effects of sport on bone mineral content independent of maturity status. Among 24 pre- or peri-pubertal tennis players of both sexes 7–13 years of age, training time was the primary predictor of inter-arm differences in bone mineral content [34]. Unfortunately, the pubertal status was assessed with “Tanner stages” without specifying the details. Moreover, given the broad CA range of the sample, CA per se is a major confounder, i.e., pre- and peri-pubertal of the same CA likely vary in body size and composition.

The present study has several limitations. The study was limited to 97 male tennis players spanning late childhood through adolescence, and of relevance, the Fels method utilizes CA- and sex-specific bone indicators [8]. As such, observations about the assessments of the most critical bones cannot be generalized. Future studies evaluating the Fels protocol should include individuals with younger CAs and also samples of the general population with equivalent distributions of skeletal maturity status (delayed, average, advanced) across adolescence.

There is a need for further study of intra-observer reproducibility and inter-observer agreement in estimates of SA among female tennis players and of other athletes as well as in the general population, and the potential impact on the distribution of youth by skeletal maturity categories within CA groups. Although the concordance between observers in classifications by skeletal maturity categories was acceptable in the present study, the sample was limited to male tennis players 8 through 16 years. A potential confounding factor is differential selection practices and persistence within specific sports which may influence the distribution of players by maturity status. For example, late maturing females tend to persist in artistic gymnastics, while early maturing males tend to persist in ice hockey, soccer and swimming [15].

Biological maturity status is implicit in models of talent identification, selection, and development. Estimates of height growth velocities over relatively short intervals in an effort to estimate the status of youth athletes relative to interval of peak height velocity are central to the long-term athlete development (LTAD) model in an effort to optimize readiness and trainability and to reduce the risk of injury [35]. Data addressing estimated velocities of growth in height relative to observed ages at peak height velocity among youth athletes, however, are lacking. In addition, maturity status classifications based on two increasingly used estimates of maturity status, predicted maturity offset (time before age at PHV) and percentage of predicted adult height at the time of observation, are at best only moderately correlated with classifications based SA in male and female tennis players [36] and on SA and stage of pubic hair in male soccer players [27]. And, predicted ages at PHV were not consistent with observed ages at PHV in female artistic gymnasts [37] and male soccer players [38–40], consistent with observations based on several longitudinal series of the general population [41–43].

Errors in Fels SA assessments associated to the observer tend to be modest and are concentrated in carpals and metacarpals. The preceding is relevant for training potential examiners. Evaluation of the reliability of assigned SAs is essential and formal training prior to SA assessments for research and perhaps clinical evaluation is essential. Inter-observer quality control is essential for studies involving more than one examiner. And, for the purpose of age verification in general and specifically in youth sports, decision-makers need to be aware of variability associated with examiners and of course method of SA assessment [15].

## Conclusions

Fels SA assessments were highly reproducible and showed an acceptable level of inter-observer agreement between trained examiners. Classifications of players by skeletal maturity status based on assessments of the two observers were also highly concordant, though not perfect. However, the impact of variation within an individual observer or between observers on maturity status classifications based on Fels SAs is relatively small and does not substantially affect the classification of youth sport participants as late (delayed), average (on time), or early (advanced) using an SA minus CA band of ± 1.0 year. Overall, the results highlight the importance of experienced observers in skeletal maturity assessments.

## Supplementary Information

**Additional file 1.**

## Data Availability

All data generated or analysed during this study are included in this published article and its supplementary information files.

## Abbreviations

SA: Skeletal age
CA: Chronological age
TEM: Technical error of measurement
%CV: Coefficient of variation
ICC: Intra-class correlation coefficient
RWT: Roche Wainer-Thissen
TW: Tanner-Whitehouse
GP: Greulich-Pyle
RUS: Radius, ulna and short bones
